# Natural history of the parasite *Waddycephalus* in the Townsville region of north-east Australia

**DOI:** 10.1017/S0031182023000197

**Published:** 2023-05

**Authors:** Halvard Aas Midtun, Megan Higgie, Conrad Hoskin

**Affiliations:** College of Science & Engineering, James Cook University, Townsville, Queensland 4811, Australia

**Keywords:** Australia, endoparasite, gecko, parasite, *Waddycephalus*

## Abstract

*Waddycephalus* is an understudied genus of pentastomids native to Australia and south-east Asia. The genus was recognized in 1922 but there has been little research on these pentastomid tongue worms over the last century. A few observations suggest a complex life cycle through 3 trophic levels. We aimed to add knowledge to the *Waddycephalus* life cycle in woodland habitats in the Townsville region of north-east Australia. We used camera trapping to identify the most likely first-intermediate hosts (coprophagous insects), we conducted gecko surveys to identify multiple new gecko intermediate host species and we dissected road-killed snakes to identify additional definitive hosts. Our study paves the way for further research into the intriguing life cycle of *Waddycephalus*, investigation of spatial variation in prevalence and impacts of the parasite on host species.

## Graphical abstract

**Figure:**
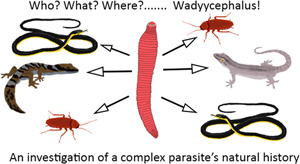

## Introduction

Pentastomids, or ‘tongue worms’, are a group of trophically transmitted parasites (Riley and Self, 1981). They are the oldest parasites known to science, first appearing in the fossil record some 500 million years ago (Paré, 2008; Siveter *et al*., 2015). However, the life cycles of most extant species are poorly known. Around 90% of extant pentastomids include carnivorous reptiles as their definitive host, while their intermediate hosts are usually unknown (Riley and Self, 1981; Paré, 2008; Barton and Morgan, 2016). The identified intermediate hosts include reptiles, amphibians, fish and insects (Lavoipierre and Lavoipierre, 1966; Kelehear *et al*., 2014; Barton and Morgan, 2016). Where information is lacking regarding the intermediate host(s), potential host species have been assumed based on the diet of definitive and intermediate hosts.

*Waddycephalus* is a Pentastomid genus within the order Porocephalida. *Waddycephalus* species are known only from Australia and south-east Asia (Riley and Self, 1981; Kelehear *et al*., 2014), and were first recognized with the description of *Pentastoma teretiusculum* (Baird, 1862), which was subsequently revised to *Waddycephalus teretiusculus* by Sambon (1922). Riley and Self (1981) conducted a morphological revision of the genus and described an additional 8 species. Seven of the 9 species are known from Australia, with records in the Northern Territory, Queensland, New South Wales, South Australia and Tasmania. Two of the *Waddycephalus* species that Riley and Self (1981) described were from south-east Asia – 1 from the Komodo Islands, where specimens were taken from a single snake (*Dendrelaphis pictus*); and the other from Hong Kong, represented by a single specimen extracted from the snake *Elaphe radiata*. Based on the sparsity of these records, the genus probably has a substantially larger distribution in south-east Asia and islands of the Pacific (Riley and Self, 1981). A recent genetic study by Kelehear *et al*. (2014), in the Darwin region, suggested higher species diversity than described from morphology. Three mitochondrial lineages conformed to recognized morphological species, but the genetic data suggested 2 additional, undescribed species. Their work, which was geographically localized, suggests that there may be substantial undescribed species diversity in *Waddycephalus*.

**Fig. 1.**
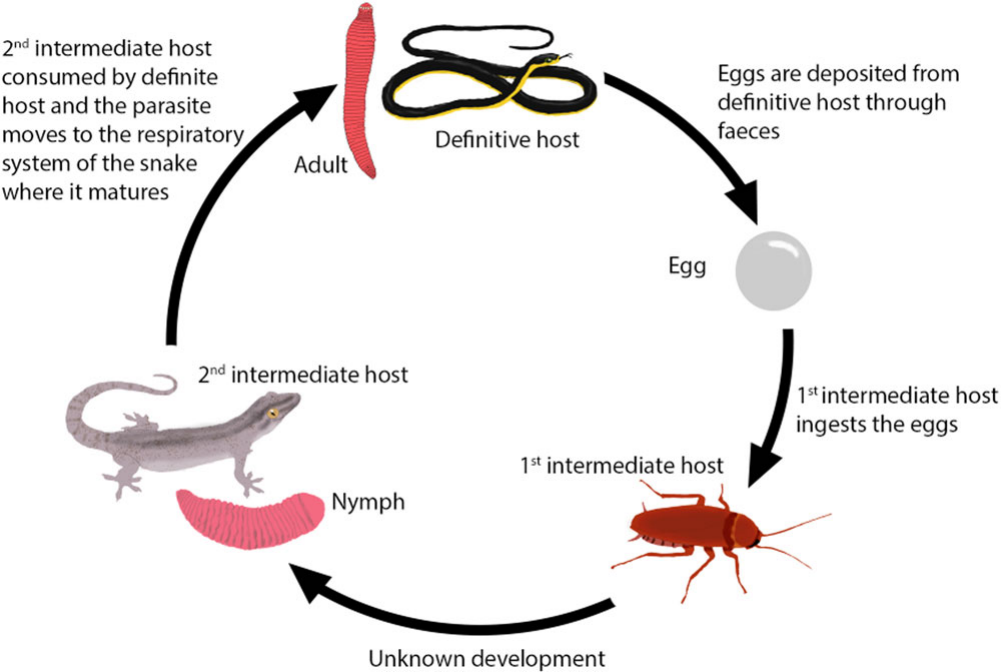
Simplified overview of the *Waddycephalus* parasite's life cycle. The 1st intermediate host step is poorly known.

*Waddycephalus* are one of the many pentastomid genera where multiple aspects of their life cycle remain poorly resolved. Based on current data and deduction, *Waddycephalus* have a 3-stage life cycle (Fig. 1). The definitive host has been confidently identified as snakes. Adult *Waddycephalus* occur inside the respiratory system of snakes (Riley and Self, 1981; Kelehear *et al*., 2014). Prior to our study, the following species were known to be potential definitive hosts through containing *Waddycephalus* in their lungs: *Acanthophis praelongus*, *Aspidites melanocephalus*, *Austrelaps superbus*, *Demansia psammophis*, *Demansia vestigiata*, *Dendrelaphis calligastra*, *Den. pictus*, *Dendrelaphis punctulatus*, *E. radiata*, *Morelia spilota*, *Notechis scutata*, *Pseudechis porphyriacus*, *Pseudonaja mengdeni*, *Pseudonaja textilis*, *Stegonotus australis* and *Tropidonophis mairii* (Riley and Self, 1981; Kelehear *et al*., 2014).

The second-intermediate host has been identified as the prey of snakes. In tropical Australia, the second-intermediate host has been primarily identified as geckos, including *Gehyra dubia*, other *Gehyra* species and *Hemidactylus frenatus* (Kelehear *et al*., 2014; Barnett *et al*., 2018), but also small mammals (Kelehear *et al*., 2014) and frogs (Kelehear *et al*., 2014). In geckos, encysted *Waddycephalus* nymphs have been identified from subcutaneous lumps (Barnett *et al*., 2018). The nymphs have also been found in subcutaneous lumps in frogs and mammals (Kelehear *et al*., 2014), but in mammals they have also been identified from intestinal connective tissue, mesentery, liver and the anal gland (Kelehear *et al*., 2014). Nymphs have been recorded once from a bird, where they were extracted from the mesentery (Kelehear *et al*., 2014).

The first-intermediate host has not yet been identified, but Riley and Self (1981) suggested that coprophagous insects (e.g. cockroaches) would be a likely first-intermediate host for *Waddycephalus* because they may consume *Waddycephalus* eggs when they feed on snake feces. However, nothing is known of this, not even the range of insects that will consume snake feces in any region.

Therefore, in the *Waddycephalus* life cycle, snakes are thought to be the definitive host (with the adults living in the lungs and then moving to the gut to shed eggs in the feces), then coprophagous insects possibly eating these eggs and becoming the first-intermediate host, then these infected insects being eaten by geckos and other small vertebrates (as the second-intermediate host) and then these being eaten by snakes (Fig. 1). However, resolving the life cycle broadly for *Waddycephalus*, and regionally, requires more data.

Here, we used a variety of field techniques to identify potential definitive and intermediate host species in the Townsville region of north-east Australia, an area of exceptional reptile diversity (e.g. Tingley *et al*., 2019; Chapple *et al*., 2021), with known *Waddycephalus* occurrence in native gecko species and the invasive Asian house gecko (*H. frenatus*) (Coates *et al*., 2017; Barnett *et al*., 2018).

## Materials and methods

### Potential definitive hosts: dissection of dead snakes for adult *Waddycephalus*

**Fig. 2.**
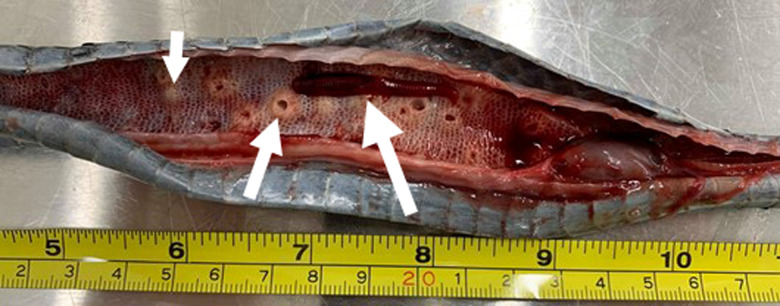
Dissected lung of a heavily infected common tree snake (*Dendrelaphis punctulatus*). It shows a mature female *Waddycephalus* sp. (largest arrow); attachment injuries from where 15 other mature parasites, removed during dissection, had anchored their heads (medium arrow); and older attachment scars (small arrow).

To inspect for adult *Waddycephalus* inside the respiratory system of snakes, dead snakes were opportunistically collected from roads in and around Townsville. A total of 33 road-killed snakes were found and dissected. The ventral scales were cut open, starting from the throat area and extending to the mid-body. The lungs and trachea were then cut open and inspected for either adult parasites or evidence of previous infections in the form of attachment scars (Fig. 2).

### Potential first-intermediate hosts: camera monitoring of snake feces

**Fig. 3.**
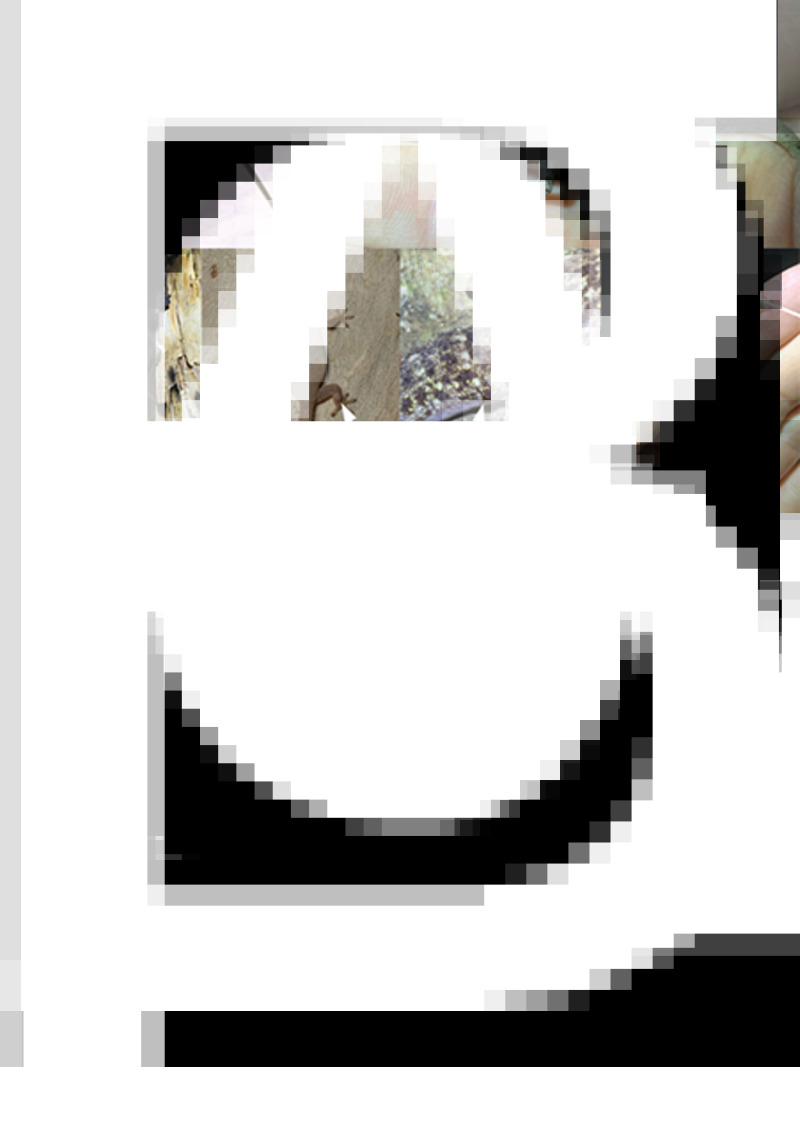
(A) *Cosmozosteria sloanei* feeding on excrements from *Morelia spilota*. (B) *Hemidactylus frenatus* caught preying on a native cockroach. (C) A heavily infected *H. frenatus* with 11 *Waddycephalus* cysts (white arrows show cysts). (D) An *H. frenatus* with a low infection of 1 cyst. (E) One of the novel host species, *Phyllurus pinnaclensis*, with 3 cysts (2 visible in the photo). (F) Another novel host species, *Amalosia rhombifer*, with 3 cysts. Photos: Halvard Midtun.

We collected snake feces from cloth bags that had been used during the relocation from houses adjacent to bushland of 4 individual snakes (which were 4 different species: *Antaresia maculosa*, *Den. punctulatus*, *Liasis fuscus* and *M. spilota*). The fecal matter was placed on the ground where the snake was released. The fecal matter was placed on a piece of white paper towel to distinguish smaller invertebrates more easily from the substrate. A camera trap (Bushnell Natureview HD Live View Trail Camera, Bushnell Corporation, Overland Park, Kansas, USA) was placed 45 cm away to monitor activity at the feces. The main targets were invertebrates such as cockroaches and other potential coprophagous invertebrates. These species are too small to trigger the motion sensor on the camera, so we set a photo interval of 1 photo every 5 min, with the camera left to record for 3 days and nights (i.e. 72 h). This gave a total of 3456 photos from 288 h of camera monitoring of the fecal deposits from the 4 snakes. These photos were manually scanned for visits by wildlife. A ‘visit’ was defined as each photo or unbroken series of photos that contained an individual of a particular species. The photos were sufficiently high resolution to assess whether the animal was feeding or just passing through (e.g. Fig. 3A).

### Potential second-intermediate hosts: field surveys for cysts in geckos

The presence of *Waddycephalus* cysts on a gecko is readily observed as subcutaneous lumps on a gecko's body, head or limbs (Fig. 3C–F). These lumps have a distinctive appearance and are known to be *Waddycephalus* cysts based on dissection of cysts on invasive and native geckos in this region (Coates *et al*., 2017; Barnett *et al*., 2018; C. H. Hoskin, unpublished data). Through a 24-month period of gecko surveys in the Townsville region, in open eucalyptus woodland, urban edge and rainforest habitats, all records of potential *Waddycephalus* cysts on geckos were obtained. Geckos were found by spotlighting with low-powered torches at night, captured gently or viewed up close without capture, and all lumps consistent with *Waddycephalus* cysts were counted.

## Results

### Definitive hosts: detection of adult *Waddycephalus* in snakes

A total of 33 dead snakes were found and dissected, representing 13 species from 3 families (Table 1). We assumed the individuals found deceased on the road were randomly hit by cars and their chances of death were not impacted by infection. Four of the 33 snakes contained *Waddycephalus* in the lungs (3 common tree snakes, *Dendrelaphis punctulata*; and 1 lesser black whip snake, *Dem. vestigiata*). One snake (a slaty grey snake, *Stegonotus cucullatus*) was not currently infected but had evidence of previous parasite activity in the form of scarred lung tissue (Table 1).

**Table 1.** Species identity of the 33 snakes in our study, and their infection details

Species	Common name	No. of dissected individuals	No. of infected individuals	Total no. of parasites found	Time of active hunting	Diet
Colubridae						
*Boiga irregularis*	Brown tree snake	3	0	0	Night	Geckos, birds
*Dendrelaphis punctulatus*	Common tree snake	7	3	25	Day	Geckos, frogs, lizards
*Stegonotus australis*	Slaty grey snake	1	0*	0	Night	Frogs, lizards, geckos
*Tropidonophis mairii*	Keelback	1	0	0	Night	Frogs, toads
Elapidae						
*Acanthophis* sp.	Death adder	1	0	0	Day/night	Lizards
*Demansia vestigiata*	Lesser black whip snake	3	1	2	Day	Lizards
*Furina ornata*	Orange-naped snake	1	0	0	Night	Lizards
*Oxyuranus scutellatus*	Coastal taipan	1	0	0	Day	Mammals
*Pseudechis australis*	Mulga snake	2	0	0	Day/night	Reptiles
*Pseudonaja textilis*	Eastern brown snake	9	0	0	Day	Mammals, reptiles
Pythonidae						
*Antaresia maculosa*	Spotted python	2	0	0	Night	Mammals, geckos
*Morelia spilota*	Carpet python	1	0	0	Night	Mammals
*Simalia kinghorni*	Scrub python	1	0	0	Night	Mammals

The asterisk represents a snake that had evidence of parasite activity in the form of old attachment scars.

### Potential first-intermediate hosts: detection of insect feeding on snake feces

The snake fecal matter was visited by 25 invertebrates, 3 mammals, 3 geckos and 2 amphibians. There was an important distinction in behaviour observed in photos: feeding on the feces (e.g. Fig. 3A) *vs* passing through the photos with no indication of feeding. We first report the species observed to be feeding on the feces and then those passing through. The most common visitor to feces was native cockroaches, with all 4 feces visited, and numerous clear photos of feeding activity (e.g. Fig. 3A). A total of 52 photos contained cockroaches and these were defined as 18 separate visits. Of these, 11 were identified as *Cosmozosteria sloanei* and 7 were *Ellipsidion humerale* (Table 2). The cockroaches visited the feces for an average time of 14.4 min (averaged from the timespan of consecutive photos from all cockroach visits). The next most common visitors to the feces were crickets (*N* = 4) and flies (*N* = 3) (Table 2), which were also observed feeding on the feces. Three rats (identification to species not possible from the photos), 3 Asian house geckos (*H. frenatus*), an ornate burrowing frog (*Platyplectrum ornatum*) and a cane toad (*Rhinella marina*) were also recorded on the cameras (Table 2) but were not observed feeding on the feces; rather, they appeared to be caught passing through.

**Table 2.** Summary of the animals recorded in camera trapping of snake feces

Taxa	Common name	No. of individuals	Behaviour observed
Observed feeding on feces			
*Cosmozosteria sloanei*	Fringed cockroach	11	Feeding
*Ellipsidion humerale*	Bush cockroach	7	Feeding
Order Diptera	Fly	3	Feeding
*Salmanites* sp.	Cricket	2	Feeding
Family Gryllidae	Cricket	1	Feeding
Observed in photo but not feeding on feces			
*Hemidactylus frenatus*	Asian house gecko	3	Passing
*Rattus* sp.	Rat	3	Passing
*Rhinella marina*	Cane toad	1	Passing
*Platyplectrum ornatum*	Ornate burrowing frog	1	Passing
Family Gryllidae	Cricket	1	Passing

The top panel shows those observed feeding on the feces in the photos and the bottom panel those only observed passing through photos.

### Potential second-intermediate hosts: detection of cysts in gecko species

Probable *Waddycephalus* nymphs (detected by characteristic subcutaneous lumps) were found in 69 of 840 geckos observed in the 24-month survey period (Table 3). These infected geckos included 5 species, from 3 families (Gekkonidae, Diplodactylidae and Carphodactylidae) (Table 3). Two of the species are known host species for *Waddycephalus*: dubious dtella (*G. dubia*) and Asian house gecko (*H. frenatus*) (Table 3; Fig. 3C and D). The other 3 gecko species are potential intermediate hosts for *Waddycephalus*: zigzag velvet gecko (*Amalosia rhombifer*) (Fig. 3F), pinnacles leaf-tailed gecko (*Phyllurus pinnaclensis*) (Fig. 3E) and Mt Elliot leaf-tailed gecko (*Phyllurus amnicola*).

**Table 3.** Gecko species examined for cysts, and details of infection status

Species	Common name	No. of individuals examined	No. of infected individuals	Proportion of infected individuals (prevalence)	Total no. of cysts observed	Median no. of cysts/infected individuals
Carphodactylidae						
Phyllurus amnicola*	Mt. Elliot leaf-tailed gecko	3	1	0.33	1	1.00
Phyllurus pinnaclensis*	Pinnacles leaf-tailed gecko	2	2	1.00	5	2.50
Diplodactylidae						
Amalosia rhombifer*	Zigzag velvet gecko	9	2	0.22	4	2.00
*Oedura castelnaui*	Northern velvet gecko	18	0	0.00	0	0.00
Gekkonidae						
*Gehyra dubia*	Dubious dtella	429	37	0.09	51	1.57
*H. frenatus*	Asian house gecko	370	27	0.07	71	2.59
*Heteronotia binoei*	Bynoe's gecko	5	0	0.00	0	0.00
*Strophurus williamsi*	Eastern spiny-tailed gecko	4	0	0.00	0	0.00

Asterisks represent novel gecko host species.

Most records came from the native species *G. dubia* and the invasive species *H. frenatus*. These are incomparably the 2 most common geckos in woodlands and urban areas in the Townsville region (Table 3; Barnett *et al*., 2017). The 2 species are morphologically and ecologically similar, and increasingly co-occur as *H. frenatus* spreads into bushland areas around Townsville (Barnett *et al*., 2017, 2018). We found low infection prevalence, overall, in *G. dubia* (9%) and *H. frenatus* (7%) (Table 3). Most individuals of these 2 species had an infection load of 1 cyst (Table 3), but individual *G. dubia* had up to 7 cysts and *H. frenatus* had up to 12 cysts. Infection rates for other species were difficult to assess due to small numbers of geckos found during the surveys (Table 3). One other observation during the field surveys that was worth noting is that multiple individuals of *G. dubia* and *H. frenatus* were observed feeding on the postulated first-intermediate hosts: cockroaches and crickets (e.g. Fig. 3B).

## Discussion

Adult *Waddycephalus* were found in 2 species of snakes, *Den. punctulatus* and *Dem. vestigiata*, with evidence of parasites in a third, *S. australis*. All 3 of these are previously recorded hosts of *Waddycephalus* (Riley and Self, 1981; Kelehear *et al*., 2014). These added records, and our lack of records in the other snake species we examined, support the evidence to date that *Waddycephalus* are found in snake species whose diet consists primarily of geckos and other lizards, and frogs (Table 1). Our infection rates are lower than in a previous study conducted in the Northern Territory by Kelehear *et al*. (2014). For example, our infection rate for the common tree snake was 42% (*N* = 7), compared to 78% (*N* = 14) in their study. However, small sample sizes in both studies make the true infection rates difficult to understand.

Cockroaches have been suggested as potential first-intermediate hosts for *Waddycephalus* (Riley and Self, 1981; Kelehear *et al*., 2014), and our results support this with the first observations of cockroaches feeding on snake feces. Of the invertebrates observed feeding on the snake feces, 75% were cockroaches (Table 2). The 2 cockroach species we observed are species native to the area. Although our observations do not prove that cockroaches ingested *Waddycephalus* eggs in the feces, these cockroaches were clearly consuming parts of the feces, which suggest that it is likely. Also supporting cockroaches as being probable first-intermediate hosts is that they are abundant in bushland in the Townsville region and are regularly observed being preyed upon by both *H. frenatus* and *G. dubia* (e.g. Fig. 3B). Of the other invertebrates observed feeding on the feces, crickets may also potentially act as hosts, but probably to a lesser degree because their diet mostly consists of living plant material (Gleeson, 2016). In contrast, the dipteran flies we observed feeding on the feces can probably be ruled out as being first-intermediate hosts because their feeding style (bubbling; Gleeson, 2016; Gomes *et al*., 2018) may not be suitable for ingesting *Waddycephalus* eggs from the feces.

Our inspection of 840 geckos in the Townsville region found that the 2 most common species, *G. dubia* and *H. frenatus*, show evidence of probable *Waddycephalus* infection, with prevalence estimates of about 9 and 7%, respectively (Table 3). This is approximately similar to the previous infection prevalence results reported for the invasive host species in this area (0–21% in Coates *et al*., 2017; 0–14% in Barnett *et al*., 2018). Interestingly, infection prevalence in these 2 hosts is similar despite *G. dubia* being native and *H. frenatus* being introduced. Three new intermediate host species were recorded in our study – 2 carphodactylid geckos (leaf-tailed geckos of the genus *Phyllurus*) and 1 diplodactylid gecko (Table 3). Infection prevalence in all 3 of these host species was higher than that for *G. dubia* and *H. frenatus* (22–100%) but very few individuals were examined (*N* = 2–9) (Table 3).

Our results add information regarding the life cycle of *Waddycephalus* but the key question remains is in identifying the species of *Waddycephalus*. We did not genetically identify the *Waddycephalus* species in our study. Our assessment of the adult *Waddycephalus* specimens we collected from snake lungs, against the descriptions of Riley and Self (1981), suggests they are *Waddycephalus longicauda* and *Waddycephalus punctulatus*. The male and female collected from the *Dem. vestigiata* have morphological features aligning with *W. longicauda*: 44–51 mm, 54–58 annuli and 8 post-vaginal annuli for females, and 14–15 mm and 55–60 annuli for males. Specimens found in *Den. punctulatus* corresponded to the description of *W. punctulatus*, having an average length of 27 mm and average annuli counts of 55, with the post-vaginal annuli count averaging 7. However, our records may involve more than the 2 species and further research should use genetic sequencing to assess local *Waddycephalus* diversity (and, if more than 1 species, host specificity), combined with morphological assessment to work out whether these species can be assigned to those already described.

In addition to genetically identifying *Waddycephalus* specimens in definitive (i.e. snakes) and second-intermediate hosts (e.g. geckos), future research should also use genetic metabarcoding or targeted amplification of *Waddycephalus* DNA from extractions of body parts from potential first-intermediate hosts. Similar techniques have been successful on other obscure parasites (e.g. Lipoldová *et al*., 2010; Huver *et al*., 2015; Polinski *et al*., 2015). This would confirm whether cockroaches do indeed ingest *Waddycephalus* eggs, and where these then develop following hatching within the host.

## Data Availability

The authors confirm that the data supporting the findings of this study are available within the article. If further details regarding each individual observation, like time, date, SVL of dissected specimens etc., is warranted the data are available from the corresponding author, H. A. M., upon reasonable request.
